# Supplementary material for: Assessing vulnerability for future Zika virus outbreaks using seroprevalence data and environmental suitability maps
Source: PLoS Negl Trop Dis. 2024 Mar 22;18(3):e0012017. doi: 10.1371/journal.pntd.0012017 (PMC10990225; doi:10.1371/journal.pntd.0012017)
Supplement: Attachment — Submitted filename: YRoell_ZikaMS_comments_responses.docx [file pntd.0012017.s002.docx]

Yannik Roell
Center for Global Health

Colorado School of Public Health

University of Colorado

yannik.roell@cuanschutz.edu

**Resubmission of Manuscript**

*Comments and Responses*

January 31^st^, 2024

Dear Editor,

We would like to thank you and the reviewers for continuing to improve the manuscript. The manuscript has been extensively reviewed and reworded according to the comments and suggestions provided. Detailed responses to inquiries and a description of changes made during the revision process can be found below. We appreciate your time and assistance in improving the manuscript.

Sincerely,

Yannik Roell

**General Comments:**

The data used within the manuscripts analysis has now been expanded to include publications from LILACS. We have also included from the original search papers from Asia. We decided that Web of Science was not a very good source because of its lack of focus on medical or clinical sciences. Even the inclusion of LILACS only gave us an additional 4 papers, which shows the comprehensiveness using PubMed. All numbers and figures have been updated to represent the new data – also considering that we removed papers from symptomatic populations (see reviewer 1, first comment).

**Reviewer 1**

| *Comments / Suggestions* | *Response* |
| --- | --- |
| Seroprevalence data from symptomatic individuals | Thanks for this comment. We went through all included studies and omitted those that were done in symptomatic individuals. Out of the ones that we considered eligible, we divided the data into four populations (pregnant women, community sample, blood donors, and other special populations, i.e. soldiers or prison inmates). The representativity of the populations cannot be further investigated or validated in this study. |
| Type of assay | The number of studies that use NTs has increased and the statement from the reviewer does not apply. The distribution of seroprevalence values coming from NTs has increased. If the reviewer is concerned that NT assays would lead to an under-estimation, NT assays are considered a reference standard, more specific, and therefore likely to provide lower estimates. |
| Date of seroprevalence studies | Maybe we were not clear on this. In the Methods section under Literature Search for Seroprevalence Estimates, we state that we use the highest and latest estimates that were available. In this way, we tried to make sure that we would use the best estimate reflecting the population immunity. |
| Categories | The cutoff was selected by visual inspection of the data, looking at the distribution of the data in the figure, and has changed with the new addition of data. The results section has been reworded to make clear that the cutoff values were based on the visual distribution of the data. |
| Clustering | Due to the comments from all reviewers and that there is an inherent issue with choosing the number of clusters within k-means, we have removed the clustering analysis within the manuscript and focused on the exploratory data analysis. |
| Line 131: French Guiana is in America, not Africa | Thanks for this comment. We have corrected this accordingly. |
| Line 179: “None of the explanatory variables were highly correlated with vulnerability” – define “highly”. Temperature is still statistically significantly correlated. | The description of the significance and correlation has been reworded to be clearer. |
| Lines 220-222: “available seroprevalence data may be under-reported due to the high frequency of non-symptomatic infections and that the data are based on clinically apparent infections (Duong et al., 2017).” – do you mean epidemiological data? | We have removed symptomatic and febrile patients as suggested so this line has been removed. Again, we appreciate you pointing out the need for removing studies conducted on symptomatic individuals. |

**Reviewer 2**

| *Comments / Suggestions* | *Response* |
| --- | --- |
| I think it would be more appropriate to define a broader range of literature databases and search terms to determine a more appropriate search strategy. | Thanks for this comment. We have included LILACS and due to the different focus not included Web of Science. In terms of alternative search terms, we were specifically looking for seroprevalence (by location). We did select 37 out of 139 that did not match our criteria during screening. With a broader array of search terms we fear that we would received more studies that would not match our criteria. Zika seems to be an infection that did not ‘attract’ too many seroprevalence surveys, unlike COVID-19. Reasons might be the focus of severe abnormalities in the fetus and thus pregnant women as the main group for surveillance? |
| I also feel that clarification is needed as to whether the reviewers independently screened the titles and abstracts, or if there are discrepancies in the reviewers' assessments, whether detailed methodology, such as by discussion or other third-party reviewer input, is addressed. | A more detailed description of the review process has been added into the Methods section under Literature Search for Seroprevalence Estimates. The added sentences outline the method used to screen the papers. |
| It was also unclear whether ethical approval had been obtained. | Ethical approval was not needed as we only used published data. |
| In fact, it is felt that a more robust approach to accurately assess these relationships would be to use continuous variables and (model) risk assessment along more geospatial gradients, rather than assigning categories. | Implementing a more robust modeling method would be outside the scope of the paper. We were trying to show first that there are areas at risk of a potential ZIKV outbreak. A more sophisticated method is the next step when the WHO finish updating the environmental suitability maps at a finer resolution with updated covariates for better predictions. |
| Temperature and vulnerability are described as positively correlated (r = 0.33) but are found to be very weakly correlated, population density and socio-economic status are also uncorrelated, and the high and low risk clusters do not seem to differ that much for any of the predictors, i.e. it appears very difficult to infer high environmental fitness for infection. | The analysis has been redone with the new data and described in more detail to indicate that we were trying to see if there was a reason we see differences between very high and low risk areas. Beforehand, we stratified the data based on seroprevalence but this time, we divided the data based on the environmental suitability and noticed that there were differences between the groups. This has been documented in the results section. |
| Since the authors are engaged in a review of the propagation of ZIKA and an assessment of the relationship between the driving factors, how, practically speaking, is there a tangible contribution that can be made in the future? What is the significance and inherent importance of the original findings that can be drawn from this research, rather than simply a systematic scoping review or repeated exercise to fill knowledge gaps in new areas? | This is a complicated comment. We understand this paper as a ‘first pass’ analysis. We don’t necessarily have the answer what the ‘tangible contribution’ would need to be. Having said this, we are able to show that there is a potential for ZIKV to re-emerge, by analyzing the discrepancy between seroprevalence and environmental suitability for transmission. We do believe that this could be of interest for decision makers in the countries affected. We see this contribution in the context of research around climate change that is often highlighting vulnerabilities to environmental hazards without giving immediate solutions. |
| Line 71: I am not sure this statement is true; Although I feel that the low anti-ZIKV seroprevalence in many parts of Latin America and the Caribbean could be described to some extent, it may not necessarily mean that environmental suitability for infection is high. The authors have not been able to fully assess causality. | We cannot prove that our statement is true. However, the environmental suitability matrix we used was published and is the best dataset on the subject available to date. More granular environmental suitability data would be welcomed. We agree that we cannot fully prove causality. However, the aim of this paper was to highlight a potential for vulnerability. |
| Lines 103-104: For reference, indicators other than Spearman's correlation coefficient (e.g., Pearson and Kendall) should also be evaluated to see the differences. | We have included the Pearson correlation coefficient in the text as well. |

**Reviewer 3**

| *Comments / Suggestions* | *Response* |
| --- | --- |
| In lines: 131 and 132: “Most of the locations for Africa were in French Guiana (N = 22) with the other 15 countries having between 1-8 locations represented.” French Guiana actually is in South America. | Thanks, this was a mistake on our side. We have corrected this. |
| Line 141. Defining vulnerability solely based on seroprevalence lower than environmental suitability leads to oversimplification of the results. This approach fails to identify other potential forms of vulnerability. In Figure 4, the Peruvian coastline does not exhibit environmental suitability, but it does not reflect vulnerability either. This is an error, as the presence of the vector mosquito, socio-economic conditions, temperature, and other factors are relevant in this area. Similarly, the state of Pernambuco experienced a high incidence of cases and possesses all the risk-inducing conditions. However, according to statistical analyses, it is depicted as a very low-risk area. This discrepancy raises questions about the effectiveness of the current assessment. | Thanks for this comment, which touches a point raised by reviewer 2 as well. We would be grateful for additional more granular environmental data to be available for all locations. However, this is not the case and the data set we downloaded likely represents the best data available on environmental suitability to date. We see the reviewer’s remark as a suggestion to produce better environmental data sets at a more granular scale. We have included a sentences in the limitations, representing the reviewer’s point of view**.** |
| Assuming a linear relationship may lead to inaccurate risk assessments, as the actual relationship between environmental suitability and transmission is likely more complex, potentially resulting in misallocation of resources for disease control. | Thanks, this point is well taken. Again, this is a first-pass analysis. The relationship may well be non-linear. The focus of this paper was to evaluate whether there is a large population at risk for future ZIKV transmission at all – and where the location might be. Future analysis will have to dive deeper into the relationships, maybe including more granular data on environmental suitability and encompassing additional co-factors (as requested by this and the other reviewers). |
| Table 1- Temperature -66-29C, is Mean temperature of coldest quarter. Source Fick and Hijmans (2017) correct? | This is correct. The data comes from WorldClim. |
| It is recommended that the rationale behind the exclusion of data from Asia, given the ongoing uncertainty surrounding the trajectory of the Zika Virus (ZIKV), should be thoroughly explained. | Thanks and we have now included data from Asia. |
